# Endobronchial Metastasis From Endometrial Carcinoma: A Case Report and Review of Literature

**DOI:** 10.7759/cureus.21995

**Published:** 2022-02-07

**Authors:** Mansur Assaad, Mohamed Swalih, Apurwa Karki

**Affiliations:** 1 Pulmonary and Critical Care Medicine, Guthrie Robert Packer Hospital, Sayre, USA

**Keywords:** lung carcinoma, endometrial carcinoma, endobronchial biopsy, bronchoscopy, endobronchial metastasis

## Abstract

Primary endometrial carcinoma presenting with endobronchial metastasis is quite rare. Little is known about predisposing risk factors, and the exact pathophysiologic mechanism remains unclear. The clinical presentation is non-specific, and symptoms likely vary depending on the disease burden. Proper tissue acquisition is necessary in order to differentiate between primary pulmonary malignancy and extra-thoracic malignancy presenting as metastatic disease. Although no formal guidelines regarding a standard diagnostic approach exist, flexible bronchoscopy with biopsy is generally regarded as having a high diagnostic yield depending on the extent of disease burden.

## Introduction

Although pulmonary metastases from extra-thoracic malignancies are quite common, the overall incidence of endobronchial metastasis is rare, specifically from primary endometrial carcinoma [1]. In fact, the incidence of endobronchial metastasis varies between 2% and 5% [2]. The most common primary malignancies associated with endobronchial involvement include breast, renal, and colorectal carcinomas [1]. However, there is little literature detailing the incidence of metastatic endometrial carcinoma with pulmonary metastases manifesting as endobronchial involvement. This may be in part related to bronchoscopy not being routinely performed on patients with obvious pulmonary metastatic disease [1]. Herein, we present a rare case of metastatic endometrial carcinoma with endobronchial involvement.

## Case presentation

**Figure 1:**
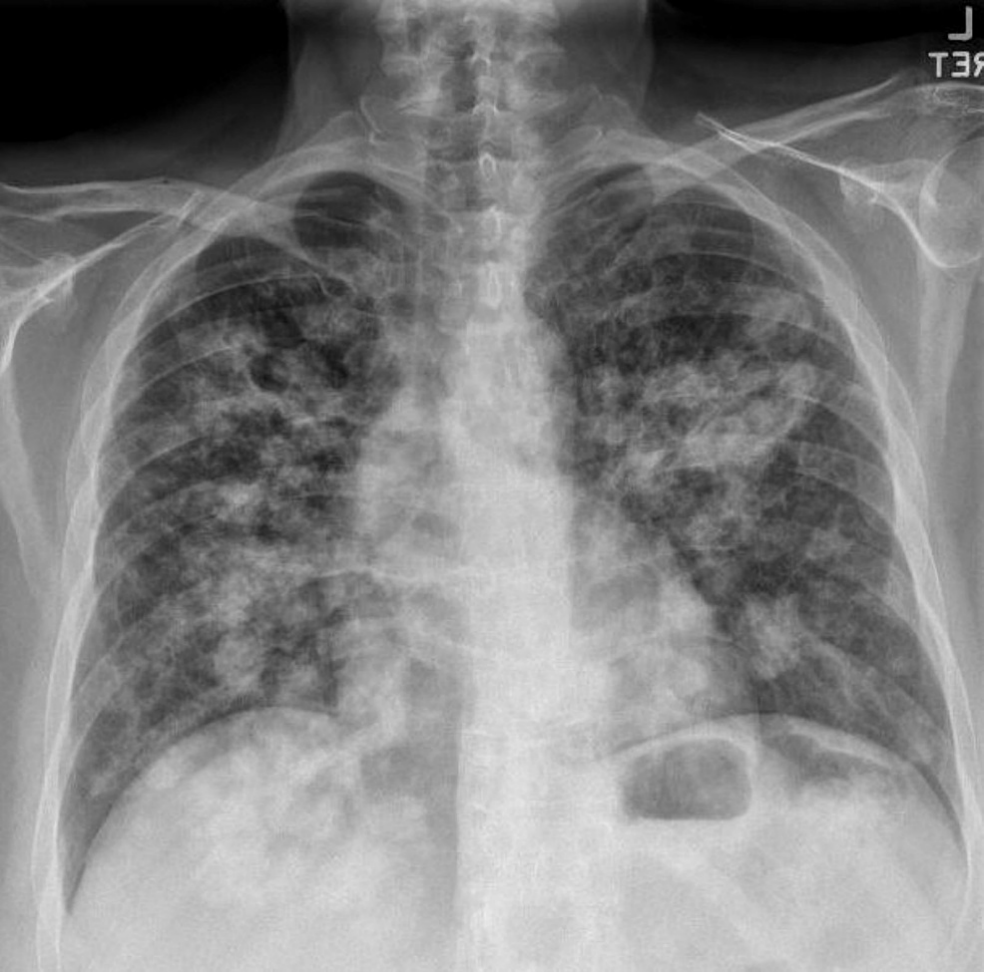
Chest X-ray showing bilateral nodular lung opacities concerning for metastatic disease

A 68-year-old woman with a history of three pack-year smoking and type 2 diabetes mellitus presented to the clinic for an abnormal chest X-ray (CXR) and computed tomography (CT) results. A CXR was performed due to persistent dry cough of one-year duration, which showed bilateral nodular lung opacities concerning metastatic disease (Figure 1).

**Figure 2:**
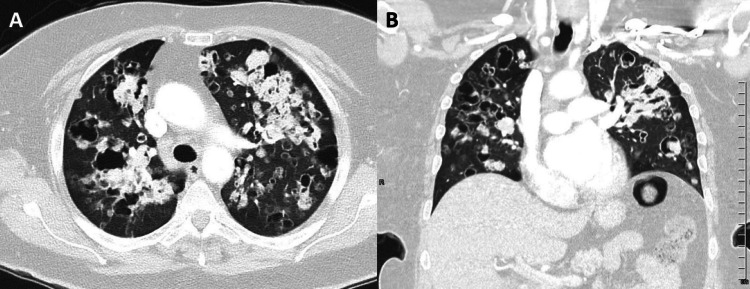
Axial (A) and coronal (B) chest CT images showing disseminated bilateral cystic, solid, and mixed solid/cystic nodules and masses with no pleural involvement

A subsequent chest CT showed disseminated bilateral cystic, solid, and mixed solid/cystic nodules and masses with no pleural involvement (Figure 2).

**Figure 3:**
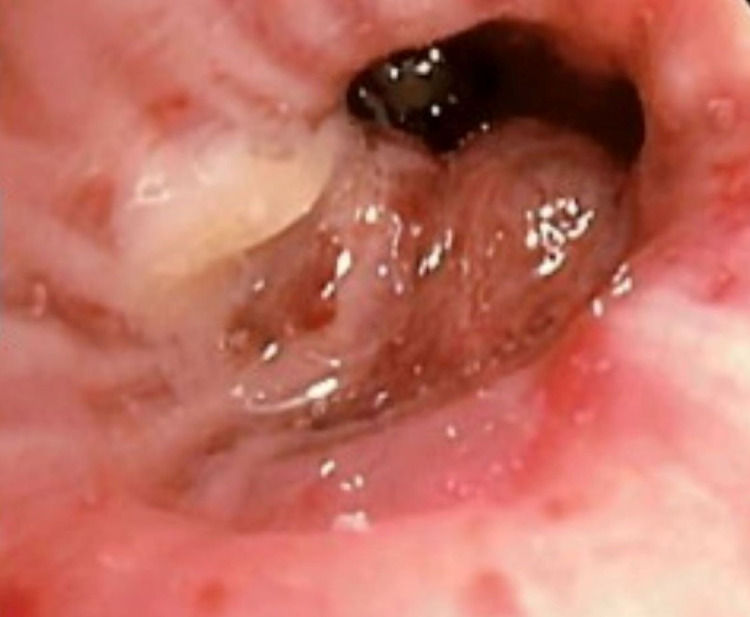
Bronchoscopy showing an endobronchial lesion partially obstructing the apical segment of the right upper lobe

No enlarged mediastinal or hilar adenopathy was present. Additionally, she endorsed post-menopausal bleeding for two years. Other than the inspiratory crackles at the left base, her physical examination was normal. Pelvic ultrasound showed an abnormally thickened uterus with a large solid soft tissue mass occupying the cervix. Subsequent positron emission tomography (PET)/CT showed extensive fluorodeoxyglucose (FDG) uptake in the uterus, abdominopelvic adenopathy, and heterogeneous pulmonary lesions. Flexible bronchoscopy revealed an endobronchial lesion partially obstructing the apical segment of the right upper lobe (Figure 3).

**Figure 4:**
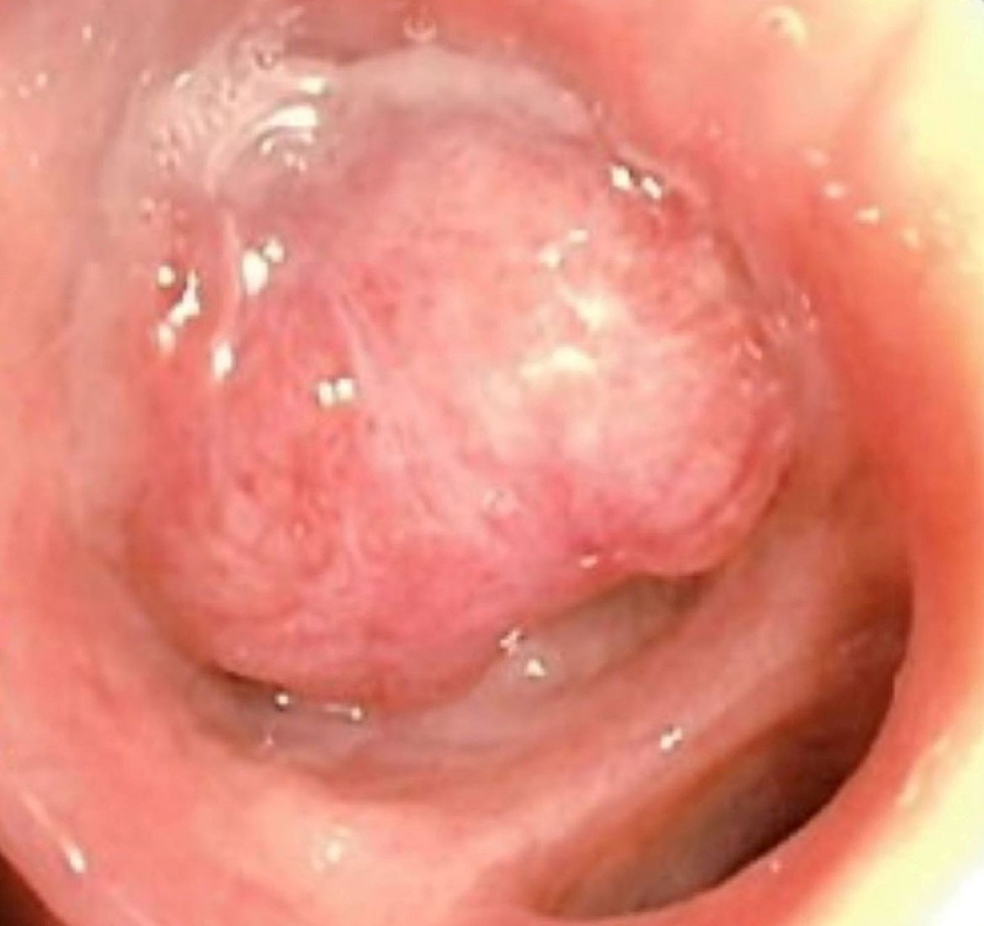
Bronchoscopy showing a large endobronchial lesion partially obstructing the right basilar segments

An additional, larger endobronchial lesion was seen partially obstructing the right basilar segments (Figure 4).

**Figure 5:**
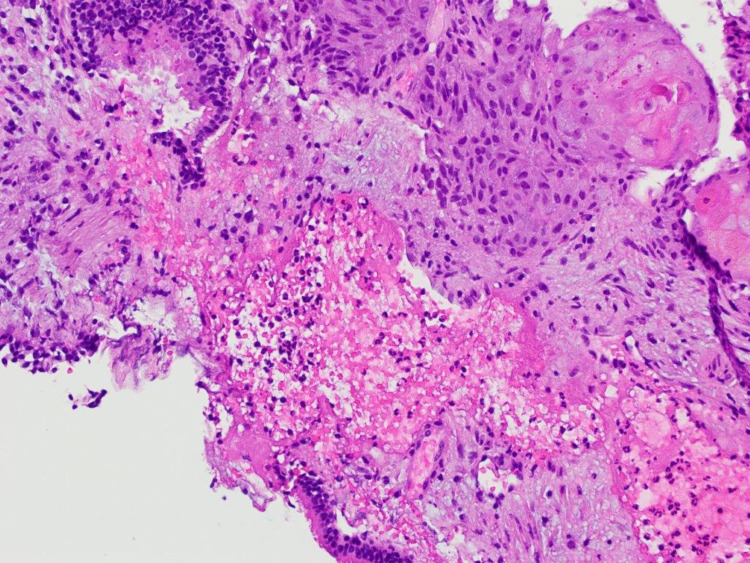
Tissue pathology from right lower lobe endobronchial lesion confirming metastatic endometrial carcinoma

**Figure 6:**
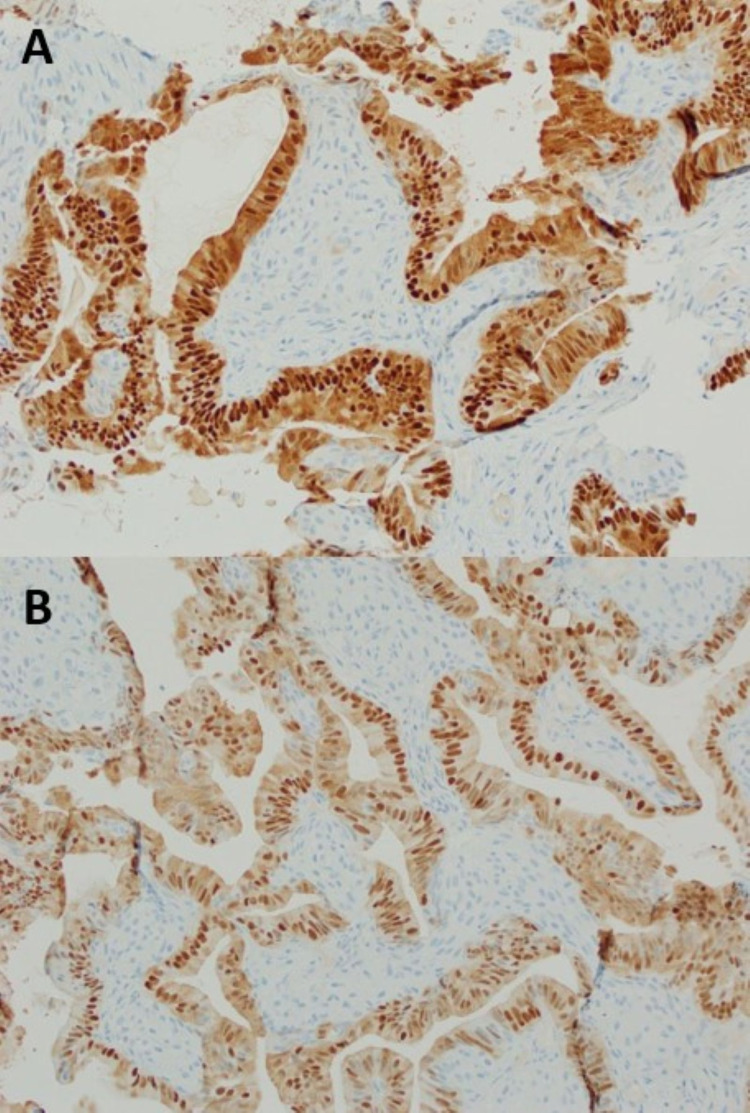
Tissue pathology of endobronchial lesion biopsy staining positive for ER (A) and PR (B)

A cryotherapy probe was introduced through the 2.8 mm working channel and used to obtain a cryobiopsy of the right basilar endobronchial lesion. Final pathology showed endometrioid glands with pseudostratified enlarged hyperchromatic nuclei surrounded by spindle cell stromal proliferation with areas of squamous pearl formation and focal keratinization, confirming metastatic endometrial carcinoma (Figure 5) staining positive for estrogen receptor (ER) and progesterone receptor (PR) (Figure 6).

## Discussion

Cryobiopsy of our patient’s endobronchial lesion confirmed the diagnosis of metastatic endometrial carcinoma. Although our patient had minimal risk factors, commonly cited risk factors for the development of endometrial carcinoma include increasing age, early menarche, late menopause, nulliparity, obesity, diabetes mellitus, and unopposed estrogen therapy [3]. Very little is known about risk factors that predispose to the development of endobronchial metastases. Additionally, the mechanism of endobronchial metastases from endometrial carcinoma is not well established and possibly includes hematogenous spread, lymphatic dissemination, or direct contiguous spread from adjacent involved tissue and lymph nodes [1,3]. Tumor biology may also play a role in the mechanism of endobronchial metastasis, and factors to consider include primary malignancy, histologic subtype, biologic characteristics, and the anatomic structures involved, particularly vascular and lymphatic involvement [1].

The clinical presentation may vary depending on how far the malignancy has advanced [4]. However, despite notable pulmonary metastases in our patient, the only respiratory symptom she displayed was a chronic dry cough. Patients may additionally present with dyspnea, chest discomfort, weight loss, night sweats, or recurrent pneumonia due to bronchial obstruction [4,5]. Imaging findings also vary depending on how far the disease has progressed [5]. CT and PET/CT remain the standard imaging modalities for characterizing disease burden. Chest CT can help confirm the presence of pulmonary metastasis and mediastinal/hilar adenopathy. However, CT may not always be able to demonstrate the presence of endobronchial lesions.

Since there may be a significant overlap between endobronchial metastases and primary lung cancer with regard to clinical presentation and radiographic findings, further evaluation with bronchoscopy is usually warranted to differentiate between the two [5,6]. No standard guidelines exist to guide bronchoscopic biopsy. Depending on the size, location, and apparent vascularity of the endobronchial lesion, standard forceps biopsy or cryotherapy can be used to obtain adequate tissue for analysis [5,6].

Between 1966 and 2002, there have been at least six documented cases of metastatic endometrial carcinoma presenting with endobronchial lesions [5,6]. After diagnosis, the median survival time was two months [5,6]. Since then, the literature review revealed only four more published, peer-reviewed case reports of metastatic endometrial cancer with endobronchial involvement [1-4]. A summary of the characteristics and diagnostic findings is displayed in Table 1.

**Table 1:** Clinical characteristics and diagnosis of five patients with metastatic endobronchial endometrial carcinoma N/A, not applicable; RUL: right upper lobe; RML: right middle lobe; RLL: right lower lobe, B/l: bilateral

Patient number	1	2	3	4	5 (our patient)
Age	73	84	43	56	68
Pertinent history	N/A	Right breast cancer	Myoma uteri	N/A	N/A
Symptoms	Abdominal pain	Dyspnea	Cough	Dyspnea, hemoptysis	Cough
Radiology	Single hypermetabolic mass	Large left lobulated mass	Multiple right-sided lung nodules	RML collapse	B/l cystic/solid masses
Location of EBM	Right lower lobe	Left mainstem bronchus	Bronchus intermedius	RML bronchus	RUL, RLL
Diagnostic procedure	Bronchoscopy	Bronchoscopy	Bronchoscopy	Bronchoscopy	Bronchoscopy
Treatment	N/A; hospice	N/A; hospice	Cryoresection, chemoradiation	Chemoradiation	Cryoresection
Reference	Lee et al. [1]	Baskaran et al. [2]	Xing et al. [3]	Hsiao et al. [4]	N/A

Among the five patients reviewed in Table 1, the age varied between 43 and 84 years. With regards to relevant prior medical history, one patient had a history of breast cancer, and another patient had uterine fibroids. Symptoms among the five reviewed patients were rather non-specific and included abdominal pain, cough, hemoptysis, and dyspnea. The location of endobronchial metastases varied and included the right lower lobe bronchus, right upper lobe bronchus, right middle lobe bronchus, bronchus intermedius, and left mainstem bronchus. All patients were successfully diagnosed with bronchoscopy and endobronchial biopsy. Interestingly, two of the patients underwent cryoresection of the endobronchial metastasis.

## Conclusions

Metastatic endometrial carcinoma with endobronchial involvement is a rare, atypical presentation that is likely underreported. Given the significant overlap of clinical symptoms and radiographic findings between primary pulmonary malignancy and pulmonary metastases, bronchoscopic evaluation with a biopsy is usually required for definitive diagnosis and proper determination of management.
